# Effect of Implicit Theory on Effort Allocation Strategies in Multiple Task-Choice Situations: An Investigation From a Socio-Ecological Perspective

**DOI:** 10.3389/fpsyg.2021.767101

**Published:** 2021-12-03

**Authors:** Keita Suzuki, Naoki Aida, Yukiko Muramoto

**Affiliations:** Department of Social Psychology, Graduate School of Humanities and Sociology, The University of Tokyo, Tokyo, Japan

**Keywords:** implicit theory, mindset, task engagement, educational environment, socio-ecological approach

## Abstract

Implicit theories refer to two assumptions that people make about the malleability of one’s ability. Previous studies have argued that incremental theorists (who believe that ability is malleable) are more adaptive than entity theorists (who believe that ability is fixed) when facing achievement setbacks. In the present research, we assumed that the adaptive implicit theory would be different when people could choose from a wider range of tasks. It was hypothesized that incremental theorists would sustain their efforts in the first task even when it was difficult, whereas entity theorists would try to find the most appropriate task. In a pair of laboratory experiments, participants had to maximize their outcomes when allowed to choose a task to engage in, from two options. When participants were allowed to practice the two tasks (Study 1), incremental theorists tended to allocate their effort solely to the first task, whereas entity theorists tended to put equal effort into both. When participants were informed that they could switch from the assigned task (Study 2), incremental theorists tended to persist in the first task regardless of its difficulty, whereas entity theorists tended to switch more quickly if the task was difficult. These results supported our hypothesis of two effort allocation strategies and implied that, in certain situations, entity theorists could be more adaptive than incremental theorists. Based on these findings, we conducted a social survey on the difficulty of switching tasks with a real-life setting as an environmental factor that determines the adaptive implicit theory (Study 3). It was revealed that the academic performance of incremental and entity theorists was moderated by the difficulty of switching tasks in their learning environment at school. Cultural differences in implicit theories may be explained by differences in the difficulty of switching tasks in education and career choices in each society.

## Introduction

How do people maintain motivation when facing difficulties in their daily lives? This has been a significant question for psychologists to answer. Previous studies have argued that an individual’s motivation toward achievement is shaped by implicit theories that are beliefs about the malleability of one’s ability (Dweck, 1986, 1999, 2006; Dweck and Leggett, 1988; Dweck et al., 1995). The belief that human attributes are malleable is called incremental theory (or growth mindset) and that human attributes are fixed is called entity theory (or fixed mindset; Dweck, 1986, 1999, 2006).

Previous studies have argued that incremental theorists are more adaptive than entity theorists during achievement setbacks (Dweck, 2006). Specifically, when facing difficulties, the former are likely to sustain their efforts toward the mastery of the task, while the latter tend to react helplessly. In this research, we assume that the adaptive implicit theory would be different when people can choose from a wider range of tasks. We predict that both theorists will adopt different strategies, which will lead to different consequences regarding their motivation and achievement. Before presenting our research perspective and hypotheses in detail, we review previous studies on implicit theories.

### Effects of Implicit Theories on Motivation in a Single-Task Situation

Dweck and Leggett (1988) described major patterns of learners’ adaptive and maladaptive behavior and proposed a model that accounts for these patterns in terms of underlying implicit theories. Incremental theorists have learning goals that make them aim for progress and improve their abilities. They tend to show “adaptive” mastery-oriented responses, such as seeking out challenging tasks and making efforts during a difficulty. However, entity theorists have performance goals that make them aim to obtain a positive evaluation of their abilities. Therefore, they tend to show mastery-oriented responses when the confidence is high, but when facing difficulty, they tend to show “maladaptive” helpless responses characterized by an avoidance of challenge and deterioration of performance.

Many empirical studies have revealed that when participants practice a task, incremental theorists practice longer than entity theorists and, therefore, get higher scores in subsequent tasks (Cury et al., 2008). Moreover, when given negative feedback, incremental theorists evoke less anxiety than entity theorists (Plaks and Stecher, 2007).

Such a trend was observed in real learning situations and laboratory settings. For instance, a longitudinal survey in a junior high school in New York showed that students with incremental theory tended to have an upward trajectory in grades in mathematics, while those with entity theory showed a flat trajectory (Blackwell et al., 2007). Hong et al. (1999) surveyed university students and found that entity theorists showed less interest in taking a remedial course even when they got a poor grade in a standardized examination. Rickert et al. (2014) reported that the stronger high school students believed in the entity theory, the more they show self-handicapping and procrastination behaviors. Nussbaum and Dweck (2008) reported that when incremental theorists fail in a test, they choose to compare their scores with the upper portion of the scale, while entity theorists compare with the lower portion of the scale to salvage their pride. Students holding the incremental theory are more resilient than those who hold the entity theory and therefore are able to buffer the negative impact of academic difficulties on their well-being (Zeng et al., 2016).

Based on these findings, researchers have reached the consensus that incremental theory is more adaptive than entity theory in learning situations (e.g., Dweck and Leggett, 1988; Aronson et al., 2002; Robins and Pals, 2002; Good et al., 2003; Plaks and Stecher, 2007). Dweck (2006) recommends that parents and teachers foster the former in students.

### Significance of Investigating a Multiple-Task Situation

Previous studies have mostly dealt with situations in which individuals engage in a specific task. This is reasonable if the main purpose of implicit theory research is to investigate the psychological process to overcome difficulties in learning situations. Dweck (2017) noted that her initial research question was: “Why do some children relish challenges and thrive in the face of the setbacks, while others who are just as skilled fear challenges and fall apart when they hit setbacks? (p. 139).” Many researchers aim to clarify the adaptive implicit theory to improve an individual’s ability to overcome difficulties and achieve a specific task.

However, in our daily lives, we often have to choose from multiple-task options that require different kinds of abilities. For example, choosing a major in college, a postgraduate plan, and a job. Considering the ubiquity of the multi-optional situations, it is also important to investigate how incremental and entity theorists behave in such situations.

We assume that when there are multiple-task options, entity theorists will not feel helpless and that the difference in strategies of the two theorists will stand out. Specifically, entity theorists, based on their belief in the fixedness of ability and performance goals, will take an aptitude exploration strategy in which they aim to choose the task they could perform best. As for incremental theorists, based on their belief in the changeability of ability and learning goals, they will take a “task mastery strategy” to improve their required ability in any task they engage in. The task mastery strategy could be inefficient in some cases of the multi-optional situation, because after choosing, they may miss the opportunity to find another task they can perform better. For example, excessively strong intrinsic motivation in a specific task reduces motivation for other tasks and the overall performance (Shin and Grant, 2019). We will discuss the positive aspects of entity theorists, which might have been dismissed in previous studies, by focusing on multi-optional situations.

A previous study indirectly supports our prediction regarding different strategies of incremental and entity theorists in multi-optional situations. Park and Kim (2015) asked participants, after working on a difficult task, to choose a follow-up task from two options. The results showed that when participants believed that a follow-up task measures the same ability as the task they failed in, incremental theorists performed better than entity theorists. However, when they believed that the follow-up task measures an ability unrelated to that need for the initial task, entity theorists showed higher performance than incremental theorists.

### Present Research

The present research aims to examine how implicit theories operate in situations involving a choice. We hypothesized that, when incremental theorists have to choose between two tasks, they will put effort solely on the first task they access and attempt to master it rather than dividing their time between the two (task mastery strategy). When entity theorists face the choice, they will try to find out which they are best suited for and will put effort to master the chosen task (aptitude exploration strategy). We tested these hypotheses through a pair of laboratory experiments, in which participants were required to maximize their outcomes when they could choose the task to engage in out of two options (Studies 1 and 2).

The task mastery strategy is effective in gaining proficiency in the selected task. However, as mentioned above, when there are multiple-task options, the learners who take this strategy may strive to master the selected task and ignore others. They may be unsure of the benefits of each task and miss the chance to achieve greater success. In this situation of multiple-task options, the aptitude exploration strategy, in which the learners explore the available information, determine which task has the most benefits, and focus their efforts on that task, is more reasonable.

This reasoning suggests that the implicit theory that leads to superior performance will be different depending on whether there are multiple options. We conducted a social survey and asked respondents about the educational environment in their middle school and how it affected their academic performance (Study 3). We hypothesize that incremental theorists perform better when they have fewer task options, while entity theorists perform better when they have more task options in school.

By considering the educational environment, we are able to discuss about implicit theories from a socio-ecological perspective. Psychological research with a socio-ecological perspective focuses on delineating how the mind and behavior are affected by socio-ecological factors, including physical, societal, and interpersonal environments (Oishi and Graham, 2010; Oishi, 2014). The core idea of this perspective is that human’s cognition, emotion, and behavior are shaped as tools for adaptation to a given environment. According to Dweck and Leggett (1988), adaptation for learners means staying motivated without feeling helpless in the face of obstacles and making an achievement (see also Elliot and Church, 1997; Elliot and Dweck, 1988). We explore what kind of implicit theory is advantageous for learners to make such an adaptation in a particular educational environment and discuss the possibility that individuals’ implicit theories are determined by the socio-ecological factors surrounding them.

There is also practical significance in considering socio-ecological factors as determinants of adaptive implicit theories. Recent meta-analyses report weak effects of implicit theories on academic achievement (Costa and Faria, 2018; Sisk et al., 2018). It has also been pointed out that educational interventions designed to induce students to develop incremental beliefs have different outcomes in different social contexts (Walton and Yeager, 2020). These suggest the importance of investigating factors that may moderate the relationship between implicit theories and academic achievement. We will hopefully provide a new perspective on the recent findings by examining the moderative effect of the educational environment.

## Study 1

Study 1 aimed to investigate the effort allocation strategies of incremental and entity theorists. Participants were presented with two tasks that measure different fictitious abilities. Then, they had to select one and perform their best. Before selecting the task, participants were provided with an opportunity to practice it. In this phase, participants were randomly assigned to one of the two tasks. The total number of practice trials was 20. The participants could switch tasks, but after doing so, they were not allowed to practice the first task again. Participants were not informed about the other task.

In such an experimental setting, we predicted that incremental theorists would adopt a task mastery strategy. Specifically, they would continue engaging in the first task longer than entity theorists to improve the ability required in the task assigned. However, entity theorists would adopt the aptitude exploration strategy and switch earlier than incremental theorists to observe both tasks. Our working hypothesis is as follows.

*H1-1*: Entity theorists switch the task earlier than incremental theorists.

Incremental theorists might aim to improve their abilities required in the first task, while entity theorists might aim to determine which task suits them by observing both tasks equally. To clarify this point, we set the following hypotheses:

*H1-2*: Incremental theorists tend to engage in the first task until the end of practice trials.

*H1-3*: Entity theorists tend to switch the task in the middle of practice trials (10 out of 20).

### Method

#### Participants

The participants were 42 Japanese undergraduate and graduate students (25 men, 17 women, *M*_Age_=21.24, SD_Age_=1.46) from the University of Tokyo. The experiment was conducted one at a time. The study was reviewed and approved by the Ethics Committee of the Department of Social Psychology, The University of Tokyo, before its commencement. The participants were informed that participation was voluntary and that they could quit at any time.

#### Procedure

##### Measuring Implicit Theory

First, each participant was presented with a questionnaire comprising three items to measure implicit theory (Hong et al., 1999) and several filler questions. The three implicit theory questions were: “You have a certain amount of intelligence, and you really cannot do much to change it.” “Your intelligence is something about you that you cannot change very much.” “You can learn new things, but you cannot really change your basic intelligence.” The Japanese translation was based on Oikawa (2005), with slight modifications. The participants answered each question on a six-point scale. A higher score indicates a stronger entity theory mindset. The filler questions comprised 10 items of the self-esteem scale (Rosenberg, 1965), and two questions on rational thinking that we composed. Responses to the filler questions were not analyzed.

##### Task Instruction

The participants were informed that the test was designed to measure their competence and that their performance would affect their reward for participation. They were then given the following briefing regarding the procedure: (1) the test features two possible tasks, one measuring “social sensitivity” and another measuring “metaphysical reasoning” (2) participants could freely choose the task to undertake, and (3) they would have an opportunity to sample both the tasks in a preliminary practice trial.

##### The Practice Trial

The participants were instructed to flip a coin to determine which task (on “social sensitivity” or on “metaphysical reasoning”) they would undertake first. However, unknown to the participants, it was predetermined that all would undertake the same task; thus, the outcome of the coin toss only determined a false label. The participants were told that the practice test would last for 20 trials. During these trials, they could switch from the first to the second task at any time, but after switching to the second task, they could not switch back. The participants were also allowed to remain in the first task. To avoid a situation where they felt that their efforts in the practice test would be wasted, the participants were advised that it would help in improving their scores in the actual test.

After the briefing, the participants undertook a practice test. The tasks were based on the Japanese version of the Remote Associates Test (RAT; Terai et al., 2013). Each question in the RAT presents three kanji characters. Although seemingly unrelated to each other, each character will form the first half of a two-character word when paired with a common fourth character. The person being tested must find the fourth character related to the three stimulus characters. Due to this design, it was unclear to the participants that the test was related to social sensitivity or metaphysical reasoning. The practice test consisted of 20 questions that exhibited a relatively low correct answer rate (<30%) in study of Terai et al. (2013) with 41 university students. This manipulation was intended to differentiate the task mastery strategy and the aptitude exploration strategy. If the first task is easy, entity theorists might continue to work on the first task because they might judge that they have an aptitude for the task, making it difficult to distinguish between the two strategies. To avoid this situation, we set the questions to be relatively difficult.

The procedure for the practice test was as follows: First, after the question number flashed on the screen for 1s, the three stimulus characters appeared for 10s. Within this time, the participants wrote down the fourth common character on the relevant field of their answer sheet. If they could not work out the answer, they left it blank. After 10s, the correct character appeared on the screen for 9s. Each question lasted for 20s. When the 20s were up, the next question appeared automatically. To enable the participants to see their progress in the first task, the screen presented the question and the number of questions completed.

The participants were instructed that they could move to the second task by pressing a key, which indicated completion. The second task was not prepared; when the participant pressed the end-task key, or if they reached 20 trials, the practice trial ended. The actual test did not take place. Finally, the participants provided their feedback in an ex post facto questionnaire. They were then debriefed and released. We recorded the number of questions that participants had engaged in (i.e., switching timing) as our main dependent variable.^1^

### Results

#### Descriptive Statistics

The reliability coefficient for the three items of the Implicit Theory Scale (6-point scale) was adequate at *α*=0.92. We averaged the scores for the three items to provide an implicit theory score (the higher the value, the stronger the entity mindset).

The average implicit theory score among the 42 participants was 3.16, with a standard deviation of 0.99, indicating that the sample leaned slightly toward the incremental theory.

#### Effects of Control Variables

First, we tested the effects of participants’ age and gender on the switching timing. Since the upper limit of the switching timing was 20, we conducted a tobit regression analysis (a model to analyze censored data) in which age and gender were independent variables, and switching timing was the dependent variable. The results indicated that age (*β*=−0.159, *p*=0.309) and gender (*β*=−0.063, *p*=0.684) did not affect switching timing. Therefore, we excluded both from further analyses.

#### Hypothesis Testing 1-1: Do Incremental Theorists Engage in the First Task Longer Than Entity Theorists?

To test hypothesis 1-1, we conducted a tobit regression analysis with implicit theory (continuous variable) as the independent variable and switching timing served as the dependent variable.^2^ Although the results were showing the trend along with H1-1, the main effect (*β*=−0.258, *p*=0.092) was not significant.

#### Hypothesis Testing 1-2, 3: Do Incremental Theorists Remain in the First Task Throughout and Do Entity Theorists Switch the Task in the Middle of the Practice Trial?

**Figure 1:**
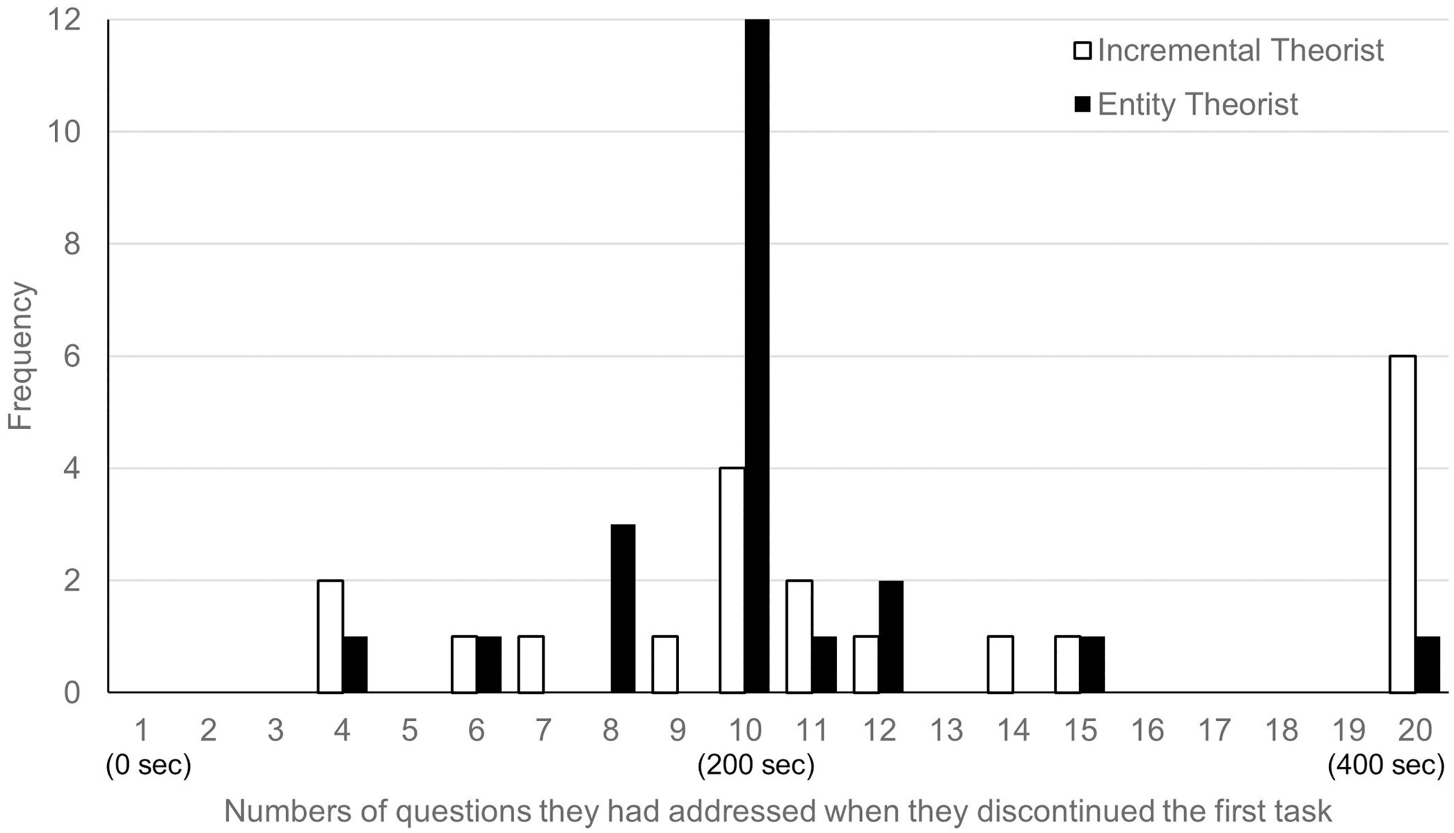
Histogram of the number of questions incremental and entity theorists had addressed when they discontinued the first task.

To visually determine the relationship between the distribution and implicit theory, we divided the participants into two groups based on their average implicit theory scores (*M*=3.16) and showed the distribution for each group (Figure 1). The figure shows that four incremental theorists, compared to 12 entity theorists, discontinued the first task at the midpoint of 10 trials. However, six incremental theorists, compared to one entity theorist, continued the task until the end. These results are consistent with our expectations.

To test hypotheses 1-2 and 1-3, we coded a new binary variable of whether the participant switched the task in the middle (no=0, yes=1) and whether the participant remained in the first task (no=0, yes=1) as dependent variables. Logistic regression analysis revealed that the more the participants held the entity theory, the less likely they were to continue the task until the end (*b*=−1.18, OR=0.31, *p*=0.027), which supports H1-2. However, there was no significant relationship between the participants’ entity belief and their likelihood of switching the task in the middle (*b*=0.67, OR=1.95, *p*=0.070). Although the latter result did not support H1-3, Figure 1 implies that entity theorists tended to switch their task around the middle of the trial. Therefore, we coded a new binary variable of whether the participant switched the task in the third quintile (i.e., whether the switching timing was from 9 to 12; no=0, yes=1) and conducted an additional analysis. Logistic regression analysis revealed that the more the participants held the entity theory, the more likely they were to switch the task in the third quintile (*b*=0.82, OR=2.26, *p*=0.027).

### Discussion

Study 1 aimed to test the hypotheses that incremental theorists adopt a task mastery strategy and that entity theorists adopt an aptitude exploration strategy. The effect of the participants’ implicit theories on their task-switching timing did not reach statistical significance, which was contrary to H1-1. However, supporting H1-2, the more the participants held incremental beliefs, the more likely they were to take the strategy of continuing the first task till the end. This implies that the incremental theorists intended to improve their ability to solve RAT. On the other hand, the participants’ entity beliefs did not predict the likelihood of taking the strategy of switching the task right in the middle of the trials, which did not support H1-3. However, the result of our additional analysis revealed that those with entity beliefs were more likely to switch the task “near the middle” of the trials. Entity theorists might have intended to observe which task was more suitable for them, although they did not predetermine dividing the opportunity to engage in each of the two tasks equally. Of course, it should be noted that the criteria we used in the additional analysis (i.e., third quintile) were somewhat arbitrary. Comprehensively, these results imply the existence of the two effort allocation strategies.

In Study 1, the participants had to make two choices: first, when and whether to switch tasks during the practice trial and second, which task to choose in the main trial. This experimental setting was useful in understanding that most entity theorists changed their tasks not because they felt helpless, but to determine their aptitude. In real life, however, we do not always have multiple options before choosing a task. We are often faced with the choice to continue the current task or to switch to a new task without complete knowledge about the new task. In Study 2, we asked participants to engage in the main trials without a practice phase and observe whether and when they would switch.

There is also a limitation. As the difficulty of the task was fixed in Study 1, we were not able to observe how entity theorists would identify their aptitude and react to the task accordingly. If they perceive their aptitude for the first task, they might continue without changing the task. Moreover, although we intended to make the task difficult to differentiate the two strategies, the difficulty might have been perceived differently between participants. It is possible that the difficulty of the task acted as an important moderator variable, leading to the weak results of Study 1. To overcome this limitation, we modified the experimental paradigm in Study 2 to compare the behavior of incremental and entity theorists with different levels of task difficulty.

## Study 2

To continue further investigation of the effort allocation strategies of incremental theorists and entity theorists, we modified the experimental paradigm of Study 1. First, in Study 2, participants engaged in the main trials from the beginning without participating in practice trials. As in Study 1, participants were assigned to one of the tasks and asked to solve them one by one. They had the choice to switch to another task. Second, unknown to the participants, there were two kinds of difficulties (Easy vs. Hard) in the first task to which they would be randomly assigned. This manipulation was designed to compare how incremental and entity theorists react when the task is difficult and when it is not. Third, we assessed the alternative explanation that entity theorists switch tasks earlier due to helplessness when faced with a difficult task.

We predicted that incremental theorists would not change their switching timing depending on the difficulty because they believe in the malleability of their ability. Therefore, they would try to improve their ability by engaging in the task regardless of the difficulty. However, entity theorists would change their switching timing depending on the difficulty because they believe in the fixedness of their ability, and, therefore, they would try to engage in the task if they can perform well. Consequently, they would engage in the first task longer when it is easy. Our working hypotheses are as follows.

*H2-1*: Incremental theorists do not change their switching timing depending on the difficulty of the task.

*H2-2*: Entity theorists engage in the first task longer when it is easy compared to when it is hard.

### Method

#### Participants

A total of 49 Japanese undergraduate students (31 men, 18 women, *M*_Age_=20.14, SD_Age_=0.71) from the University of Tokyo participated in the experiment. Since it was conducted as a part of a research method course in psychology, participants did not receive monetary reward and went through the experiment simultaneously in the same room. The study was reviewed and approved by the Ethics Committee of the Department of Social Psychology, The University of Tokyo, before its commencement. The participants were informed that participation was voluntary and that they could quit at any time. The participants were informed that the participation or the score would not affect their course grade.

#### Procedure

##### Measuring Implicit Theory

First, participants were presented with a questionnaire comprising three items to measure implicit theory (Hong et al., 1999) and several filler questions. The Japanese translation was based on Oikawa (2005), with slight modifications.^3^ The filler questions comprised 17 items of the Goal Orientation Scale (Mitsunami, 2010). Responses to the filler questions were not analyzed. The reliability coefficient for the three items of the Implicit Theory Scale (6-point scale) was adequate at *α*=0.94. We averaged the scores for the three items to provide an implicit theory score (the higher the value, the stronger the entity mindset).

##### Task Instruction

First, the participants were informed that the test was designed to measure their ability for abstract thinking and that two different kinds of tasks, one measuring their “social sensitivity” and another their “metaphysical reasoning ability” were prepared.

Next, they were instructed on how the experiment proceeds: (1) participants will receive two booklets that contain two different tasks, (2) each participant will be assigned to a task, (3) during the trials, participants will be able to change tasks anytime, but after switching, they will not be allowed to switch back (they could also remain in the first task throughout), (4) the experiment will end when they solve 20 questions, regardless of the task they choose, and (5) their grade will be calculated by the total number of correct answers in both tasks. They were encouraged to obtain as many correct answers as possible.

##### Content of the Task

Half of the participants were led to believe that their first task measured “social sensitivity,” and the other half believed that their first task measured “metaphysical reasoning ability.” However, it was predetermined that all participants performed the same task: the Japanese version of RAT (Terai et al., 2013). Although the task was identical to that used in Study 1, the difficulty was manipulated. Specifically, based on the accuracy rate reported in Terai et al. (2013), we selected the 20 highest accuracy-rate trials for the easy task and 20 lowest accuracy-rate trials for the hard one. Participants were randomly assigned to either; however, they were not informed of this.

The second task was the Japanese version of the anagram test, which was randomly selected from Aoyagi and Oashi (1990). The accuracy rate was ranged from easy to hard versions of the first task. However, the information of the second task was not provided until the participants chose to switch tasks.

##### Main Trial

After the instruction, all participants were asked to immediately start the first task. First, participants were given 60s to read the instructions of the RAT. Then, they were instructed to move on to the questions. Participants were given 15s to solve one question and 30s to check the answer, and so, each trial lasted for 45s. The timing of the page turn was dictated by the experimenter, so they could not turn it even if they solved the question or finished checking their answers within the assigned time. If participants wanted to switch their task, they had a 30-s answer-checking period to do that. The entire task ended when the total number of questions reached 20. If the participants did not switch their task, their switching timing was measured as 20.

##### Post-task Questionnaire

After the trials, the participants were asked to answer the post-task questionnaire. Several items included a measure of helplessness (“During the task, I felt helplessness”). Since the experiment was not incentivized by monetary rewards, we also measured the participants’ intention to get good scores (Performance intention: “I intended to get good scores”) as a control variable. Each item was measured using a 6-point Likert scale.^4^

### Results

#### Descriptive Statistics

Table 1 shows the mean scores and standard deviations of the main variable used in the following analysis. The average implicit theory score among the 49 participants was 4.18 with a standard deviation of 1.21, indicating that the sample leaned slightly toward the entity theory. Most participants continued the first task longer and till the end, which was the most frequent pattern.

**Table 1:** The descriptive statistics of the main variables used in Study 2.

	EASY (*N*=25)		HARD (*N*=24)		ALL (*N*=49)	
	*M*	*SD*	*M*	*SD*	*M*	*SD*
Implicit theory	4.09	1.35	4.29	1.06	4.18	1.21
Switching timing	14.64	5.05	13.67	4.46	14.16	4.74
Performance intention	4.64	1.55	4.62	1.10	4.63	1.33
Helplessness	2.08	1.32	2.71	1.55	2.39	1.46

#### Effects of Control Variables

First, we tested the effects of the participants’ age and sex on switching timing. Since the upper limit was 20, we conducted a tobit regression analysis with age and gender as independent variables, and the switching timing served as a dependent variable. The results indicated that neither age (*β*=0.186, *p*=0.266) nor gender (*β*=−0.162, *p*=0.210) affected the switching timing. Therefore, we excluded both from further analyses.

#### Hypothesis Testing 2-1, 2: How Do Incremental and Entity Theorists Behave Differently to Easy and Hard Tasks?

**Figure 2:**
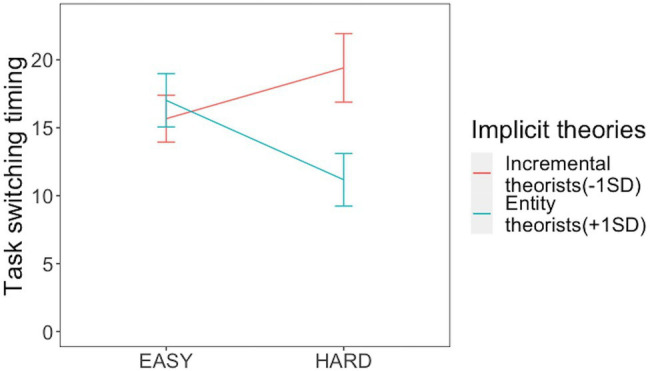
The effects of task difficulty and implicit theories on task-switching timing.

To test hypothesis 2-1 and 2-2, we conducted a tobit regression analysis with the implicit theory, difficulty of the task (Easy=0, Hard=1), and their interaction as the independent variables, with switching timing being the dependent variable.^5^ Performance intention was added to the analysis as a covariate. Neither the main effect of implicit theory (*β*=−0.244, *p*=0.110) nor difficulty (*β*=−0.075, *p*=0.590) were significant. A significant interaction between implicit theory and task difficulty was found (*β*=−0.344, *p*=0.026). Simple slope analysis^6^ (Figure 2) revealed that among incremental theorists (−1 SD), the main effect of task difficulty (*β*=0.273, *p*=0.221) was not significant. Among entity theorists (+1 SD), the main effect of difficulty of the task (β=−0.424, *p*=0.035) was significant, suggesting that entity theorists switched earlier than incremental theorists, which supported H2-1 and H2-2.

#### Additional Analysis: Did Helplessness Affect Entity Theorists?

We conducted a regression analysis with implicit theory, difficulty of the task and their interaction as independent variables, and helplessness as dependent variables. Neither the main effect of implicit theory (*β*=−0.037, *p*=0.800) nor the difficulty of the task (*β*=0.221, *p*=0.131) were significant, and the interaction of these variables was also not (*β*=0.175, *p*=0.248). This suggests that entity theorists did not switch because they felt helpless.

### Discussion

In Study 2, we tested the hypotheses that incremental theorists do not change their switching timing depending on the difficulty of the task, and that entity theorists engage in the first task longer when the task is easier. The results supported these hypotheses, indicating that entity theorists changed their reactions depending on whether they thought they had the aptitude for the task which strengthens our prediction that they tend to adopt an aptitude exploration strategy. However, incremental theorists did not change their reactions depending on the difficulty but consistently tried to face it. This result strengthens our prediction that incremental theorists tend to adopt a task mastery strategy.

Additionally, the results indicated that entity theorists’ task choice strategy was not due to their helplessness in a difficult task. There was no difference between incremental and entity theorists in their evoked helplessness. This is different from the findings of previous studies (e.g., Dweck and Leggett, 1988), which emphasized the helplessness felt by entity theorists when faced with difficulty. They might not feel helpless when there are alternative task options because they can utilize their aptitude exploration strategy. The difference between the present research and most previous studies comes from the presentation of different situations in which there are alternative task options.

It should be noted that, in Study 2, we were not able to evaluate whether each of the two strategies was adaptive or not based on the performance of the participants. This was because the results would vary arbitrarily depending on how the researcher set the difficulty level of the second task. Also, although Dweck and Leggett (1988) assumed that leaners who can make adaptive responses in the face of difficulty would have higher achievement, it would not necessarily be seen in an individual test score, but rather, would appear in more comprehensive academic performance. Therefore, in Study 3, we conducted a social survey and asked participants to indicate their academic performance, which would reflect the consequence of their cumulative use of the task mastery or aptitude exploration strategies.

## Study 3

In Study 1 and 2, we investigated effort allocation strategies in a multiple-task situation. The results implied that incremental and entity theorists adopt the task mastery strategy, and the aptitude exploration strategy, respectively. Additionally, Study 2 suggested that when there are multiple-task options, entity theorists could avoid helpless responses as well as incremental theorists. Comparing these results with previous studies on a single-task situation shows that preferable implicit theory may depend on whether task-switching is possible in a learning environment.

A previous study claimed that when there is only one task, incremental theorists would be more adaptive because they can utilize their task mastery strategy, while entity theorists are unable to use the aptitude exploration strategy because they have no other task to explore. However, when there are multiple-task options, incremental theorists might stick to a difficult task (as seen in Study 2), which might cause opportunity costs. In Study 3, we aim to investigate how environmental factors related to task-switching moderate the advantage of incremental and entity theories in real-life settings.

In Studies 1 and 2, we dealt with the factor as a binary variable (i.e., a single or multiple-task situation) in a laboratory setting. In Study 3, we focus on the task-switching difficulty in a learning environment, which is defined by the number of task options, the external force to make individuals engage in a specific task, and the cost of switching.

School education, including school policies and curricula, might provide a good example. In Japan, some schools force all students to work on a standard curriculum, while other schools allow individual students to choose courses and subjects. In the former, students who have fallen behind in their studies often face pressure to catch up with the majority in the specific curriculum. We assume that students with incremental theory would be better at achieving in such an environment. However, in schools with a flexible curriculum, students with entity theory may utilize their aptitude exploration strategy and achieve results without feeling helpless.

To investigate the moderating effect of the task-switching difficulty, we conducted a social survey that asked the participants’ educational experience. We used the uniformity of education of the participants as an indicator of task-switching difficulty and their academic performance as an indicator of the consequence of having a specific implicit theory. The working hypotheses are as follows.

*H3-1*: When the uniformity of education in school is high, students with the incremental theory perform better than those with the entity theory.

*H3-2*: When the uniformity of education at school is low, students with the entity theory perform better than those with the incremental theory.

Specifically, we asked respondents to recall and rate the uniformity of the classes and instructions they received at a junior high school, and examined how these measures moderate the impact of implicit theories on their academic performance. Academic performance was measured by asking respondents about their grades in junior high school and their high school level.

Prior to testing our hypotheses, we investigated two issues to confirm the findings of Study 1 and 2. First, we measured the respondents’ aptitude exploration behavior and tested the correlation between implicit theories and the behavior to confirm the ecological validity of Study 1 and 2. Second, in order to confirm that entity theorists do not show helpless responses when they have multiple-task options, we measured the participants’ satisfaction with school life and analyzed how it was affected by implicit theories and uniformity of education.

### Method

#### Participants

A total of 500 Japanese adults (250 men, 250 women, *M*_Age_=26.63, SD_Age_=2.22) who were registered as monitors of Cross Marketing Inc. participated in the survey. The participants’ age was restricted between 22 and 29years to minimize generational differences in the educational experience. The study was reviewed and approved by the Ethics Committee of the Department of Social Psychology, The University of Tokyo, before its commencement. Participants were informed that participation was voluntary and that they could quit at any time.

#### Questionnaire

Study 3 was conducted as part of a research project on the educational environment of elementary, junior high, and high schools in Japan, where the first two are compulsory, with many students taking high school entrance exams. Therefore, to test our hypotheses, we focused only on the participants’ learning environment in junior high school and used their high school’s rank as a performance indicator. Below, we specify the items used to examine the hypotheses. The details of the questionnaire and supplementary analysis are available in the Supplementary Material.

#### Implicit Theory

The participants indicated their entity or incremental beliefs that they had endorsed in their school days on a 6-point Likert scale (“In junior high school, I believed that ability is something about you that you cannot change very much”).

#### Uniformity of Education at Junior High School

The participants indicated the uniformity of education on a 6-point Likert scale (“At my junior high school, all students were expected to learn at the same pace.” or “At my junior high school, delayed learning made school life uncomfortable.” or “At my junior high school, many classes involved memorizing textbook content.”). Since the reliability coefficient for the three items was adequate (*α*=0.82), we calculated the uniformity of the class score by averaging the scores of the three items.

#### Academic Performance

The participants indicated their relative ranking of academic records within their school grade on a 5-point Likert scale. A lower number indicated a higher ranking; therefore, we reversed the score in the analysis. Those who did not specify their ranking of academic records were excluded from the analysis.

As another indicator of school performance, participants indicated their high school’s relative ranking on a 5-point Likert scale.^7^ A lower number indicated a higher ranking; therefore, we reversed the score in the analysis. Those who did not specify their high school level were excluded. The participants indicated whether they went through entrance exams or interviews. For those who did not take an entrance exam, the high school’s ranking does not necessarily indicate their academic performance. Therefore, they were excluded from the analysis.

#### Aptitude Exploration Behavior

The participants indicated the extent to which they had engaged in aptitude exploration behavior on a 5-point Likert scale (“In junior high school, I tried to find and develop my talents, not just in my studies”).

#### Satisfaction With School Life

The participants indicated the extent to which they were satisfied with their school life on a 5-point Likert scale (“Overall, I was satisfied with my junior high school experience”).

#### Demographic Variables

The participants indicated their age, gender, and their parents’ educational qualification (which was dummy coded into binary variables that indicate whether they graduated from university). Those who did not specify their parents’ educational attainment were excluded from the related analysis. Participants indicated their economic status in their school days on a 5-point Likert scale.

### Results

#### Descriptive Statistics

The descriptive statistics are listed in Table 2. Both the academic record and the high school’s ranking of participants were significantly correlated with their parents’ educational attainment and economic status. Therefore, to assess the robustness of our analysis, we tested two models, with and without covariates.

**Table 2:** The descriptive statistics of the variables used in Study 3.

	*N*	*M*	*SD*	2	3	4	5	6	7	8	9	10	11
1. Implicit theories	500	3.36	1.21	0.415^**^	−0.120^*^	−0.126^**^	0.255^**^	0.022	−0.054	−0.043	−0.012	−0.067	0.023
2. Uniformity of the class	500	3.67	0.97	–	0.009	0.012	0.280^**^	0.130^**^	0.009	−0.020	0.045	−0.003	0.031
3. Academic record	452	3.18	1.21		–	0.506^**^	0.200^**^	0.197^**^	0.194^**^	0.099^*^	0.168^**^	0.025	−0.002
4. Ranking of the high school	449	2.77	1.14			–	0.184^**^	0.276^**^	0.201^**^	0.133^**^	0.234^**^	0.065	−0.092^*^
5. Aptitude exploration behavior	500	3.15	1.20				–	0.290^**^	0.071	0.087	0.127^**^	0.005	−0.018
6. Satisfaction with school life	500	3.20	1.32					–	0.034	0.026	0.201^**^	0.071	−0.035
7. Educational attainment dummy (Father; 1=Graduated university)	410	0.52	0.50						–	0.401^**^	0.168^**^	0.050	−0.078
8. Educational attainment dummy (Mother; 1=Graduated university)	436	0.26	0.44							–	0.140^**^	0.014	−0.092^†^
9. Economic status	500	2.99	1.01								–	0.018	−0.063
10. Age	500	26.6	2.24									–	0.038
11. Gender	500	–	–										–

** *p<0.01*;

* *p<0.05*;

† *p<0.10*.

The participants’ implicit theory was significantly correlated with their aptitude exploration behavior (*r*=0.255, *p*<0.001), which implies that the stronger the participants endorsed entity beliefs, the more likely they took on aptitude exploration behavior.

#### Interaction Effect of Implicit Theories and Uniformity of Education on Satisfaction With School Life

**Figure 3:**
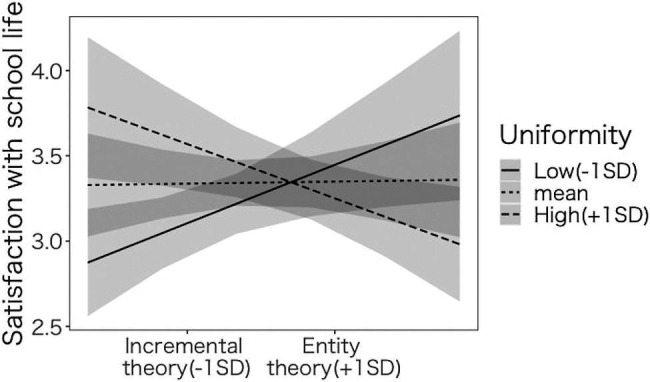
The effects of implicit theories and uniformity of education in junior high school on satisfaction with school life.

We conducted a regression analysis with satisfaction with school life as the dependent variable (Table 3). The interaction between implicit theories and uniformity of education was significant (Model 0-1: *β*=−0.133, *p*<0.001, Model 0-2: *β*=−0.161, *p*<0.001). We conducted a simple slope analysis (Figure 3). Among the participants whose uniformity of education was high (+1 SD), when the demographic variables were controlled, the main effect of implicit theories was significant (Model 0-1: *β*=−0.065, *p*<0.312, Model 0-2: *β*=−0.146, *p*<0.019), suggesting that the more the participants endorse incremental theory, the more satisfied they were with their school life with highly uniform education. Among the participants whose uniformity of education was low (−1 SD), the main effect of implicit theories was significant (Model 0-1: *β*=0.178, *p*<0.002, Model 0-2: *β*=−0.157, *p*<0.021), suggesting that the more the participants endorse the entity theory, the more satisfied they were with their school life with non-uniform education.

**Table 3:** Results from regression models on the ranking of high school and the academic records.

Independent variables	Model 0-1 Satisfaction with school life (*N*=500)		Model 0-2 Satisfaction with school life (*N*=410)		Model 1-1 Academic record (*N*=449)		Model 1-2 Academic record (*N*=410)		Model 2-1 Ranking of the high school (*N*=370)		Model 2-2 Ranking of the high school (*N*=304)		*β*	*t*	*β*	*t*	*β*	*t*	*β*	*t*	*β*	*t*	*β*	*t*
Implicit theories	−0.043	−0.89	−0.012	−0.23	−0.142	−2.84^**^	−0.124	−2.29^*^	−0.164	−2.95^**^	−0.121	−1.95^†^
Uniformity of education	0.118	1.68^*^	0.049	0.88	0.093	0.89	−0.023	−0.43	0.093	1.68^†^	0.060	0.95
Implicit theories×Uniformity of education	−0.133	−4.31^**^	−0.161	−4.31^**^	−0.094	−2.85^**^	−0.098	−2.64^**^	−0.074	−2.04^*^	−0.138	−3.12^**^
*Covariates*												
Educational attainment dummy (Father; 1=Graduated university)			0.000	0.02			0.155	2.95^**^			0.165	2.83^**^
Educational attainment dummy (Mother; 1=Graduated university)			−0.014	−0.27			−0.004	−0.09			−0.012	−0.22
Economic status			0.140	2.89^**^			0.133	2.66^**^			0.131	2.39^*^
Age			0.045	0.92			−0.023	−0.42			−0.023	−0.41
Gender			−0.036	−0.78			−0.015	−0.32			−0.125	−2.30^*^

*In Model 0-2, participants who did not specify either of their parents’ educational attainment were excluded. In Model 1-1, participants who did not specify their ranking of academic records were excluded. In Model 1-2, from the sample used in Model 1-1, participants who did not specify either of their parents’ educational attainment were excluded. Model 1-1 and 2-1 do not have covariates and Model 1-2 and 2-2 have covariates. Values are standardized coefficients with t-values. In Model 2-1, participants who indicated that they did not experience an exam or an interview and those did not specify their high school’s ranking were excluded. In Model 2-2, from the sample used in Model 2-1, participants who did not specify their parents’ educational attainment were excluded*.

** *p<0.01*;

* *p<0.05*;

† *p<0.10*.

#### Hypothesis Testing 3-1, 2: Does Uniformity of Education in Junior High School Moderate the Effect of Implicit Theory on Academic Performance?

**Figure 4:**
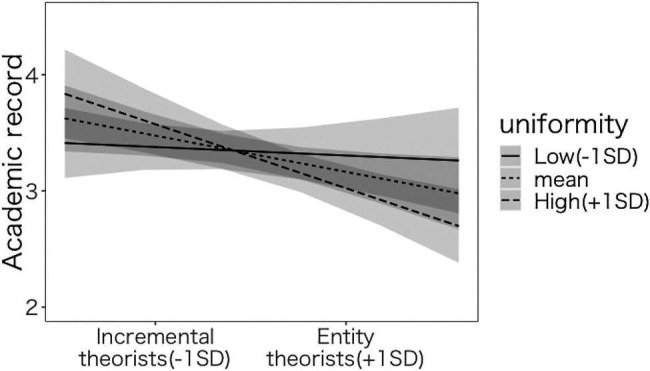
The effects of implicit theories and uniformity of education in junior high school on academic record.

To test hypotheses 3-1 and 3-2, we conducted a regression analysis with the academic record as the dependent variable (Table 3). The interaction between implicit theories and uniformity of education was significant (Model 1-1: *β*=−0.094, *p*<0.004, Model 1-2: *β*=−0.098, *p*<0.008). We conducted a simple slope analysis (Figure 4). Among the participants whose uniformity of education was high (+1 SD), the main effect of implicit theories was significant (Model 1-1: *β*=−0.237, *p*<0.001, Model 1-2: *β*=−0.220, *p*<0.001), suggesting that the more the participants endorse the incremental theory, the higher the relative rank of their academic record. Among the participants whose uniformity of education was low (−1 SD), the main effect of implicit theories was not significant (Model 1-1: *β*=−0.048, *p*<0.433, Model 1-2: *β*=−0.032, *p*<0.640).

**Figure 5:**
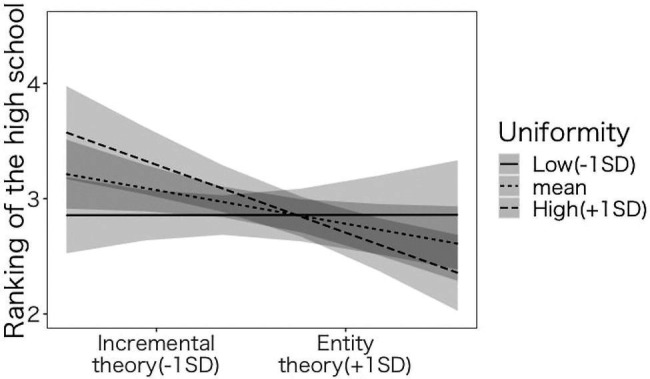
The effect of implicit theories and uniformity of education in junior high school on ranking of the high school.

We conducted a parallel analysis with the high school’s ranking as the dependent variable (Table 3). Both models, with and without covariates, revealed that the interaction between implicit theories and the uniformity of the class was significant (Model 2-1: *β*=−0.164, *p*<0.003, Model 2-2: *β*=−0.138, *p*<0.001). We conducted a simple slope analysis using Model 2 (Figure 5). Among the participants whose uniformity of education was high (+1 SD), the main effect of implicit theories was significant (Model 2-1: *β*=−0.239, *p*<0.001, Model 2-2: *β*=−0.257, *p*<0.001), suggesting that the more the participants endorse the incremental theory, the higher the high school’s ranking, which supported H3-1. Among the participants whose uniformity of education was low (−1 SD), the main effect of implicit theories was not significant (Model 2-1: *β*=−0.089, *p*<0.180, Model 2-2: *β*=−0.001, *p*<0.989). This does not support H3-2. An identical pattern was obtained with the analysis in which the academic record and the ranking of the high school served as the dependent variable, which supports only H3-1.

## Discussion

Study 3 aimed to investigate how adaptive implicit theory is determined by the task-switching difficulty in a learning environment. To achieve this goal, we conducted a social survey measuring the uniformity of education at school as an indicator of task-switching difficulty and academic achievement as an indicator of performance.

The results supported H3-1, suggesting that in an environment where task-switching is difficult, endorsing incremental theory is more adaptive. However, the results did not support H3-2 but suggested that in an environment where task-switching is easy, incremental and entity theorists performed to the same extent. Although the results did not show the advantage of entity theory in an environment where task-switching is easy, it is important to know that entity theorists can achieve the same level of achievement as incremental theorists depending on the educational environment. It should be noted that our hypotheses were based on the assumption that individuals with entity beliefs were more likely to engage in aptitude exploration behavior than those with incremental beliefs; this correlation was confirmed in Study 3, consistent with the results of Study 1 and 2. Moreover, individuals with entity beliefs tended to be more satisfied with their school life in a non-uniform educational environment, while those with incremental beliefs showed the opposite tendency. This result, along with the findings from Study 2, suggests that entity theorists’ feeling of helplessness may be reduced when alternative task options are available. Overall, the results imply that the task-switching difficulty is a boundary condition that determines the advantage of incremental and entity theories.

In future research, it is necessary to measure other aspects of the task-switching difficulty. For example, a single or multiple-track system, a variety of course curricula, and the freedom to choose your favorite subjects might be other aspects of task-switching difficulty. Entity theorists could be adaptive to the learning environment with high freedom of choice. Further investigation is needed to measure and test the effects of other dimensions of task-switching difficulty.

## General Discussion

### Summary

The study aimed to investigate effort allocation strategies of incremental and entity theorists according to the learning environment. In Studies 1 and 2, we focused on their strategies in situations with multiple choices of tasks and tried to compare the results with those of previous studies on situations with no choices of tasks. We predicted that incremental theorists, based on their belief in the malleable nature of their ability, would adopt the task mastery strategy and allocate all their efforts to master a specific task regardless of whether they had a choice. In contrast, we predicted that entity theorists, based on their belief in the fixed nature of ability, would use the aptitude exploration strategy to choose the most suitable task and then put their effort into it. To test these hypotheses, in Study 1, we provided the participants with an opportunity to sample and practice two tasks before choosing the one to engage in and observed how incremental and entity theorists switched from the assigned task. The results revealed that incremental theorists tended to practice the first task longer than entity theorists; specifically, they tended to practice the assigned task throughout. However, entity theorists tended to switch tasks in the middle of the practice. In Study 2, we observed how incremental and entity theorists switched their tasks when they could not practice and started with trials directly related to their grades. We also manipulated the difficulty of the first task. The results revealed that while incremental theorists did not change their switching timing depending on the difficulty, entity theorists engaged in the first task longer when the task was easier. Additionally, there was no difference in helplessness evoked between incremental and entity theorists, which means that entity theorists’ strategy was not driven by a feeling of helplessness, as claimed in previous studies.

Based on these findings in laboratory settings, we predicted that the choice of implicit theory would depend on whether an individual can switch tasks easily in a learning situation. In Study 3, we focused on the task-switching difficulty in a real-life setting and conducted a social survey to investigate the advantage of incremental and entity theories depending on learning environments. It was found that, in the case of respondents who had studied in junior high schools with highly uniform education, incremental theorists were more satisfied with their school life, performed better in junior high school, and also went on to higher-ranked high schools. However, in the case of those from junior high schools with more selective education styles, entity theorists were more satisfied with their school life, while there was no difference between the academic performance of incremental theorists and entity theorists. We also confirm that holding entity beliefs was correlated with aptitude exploration behavior in a real educational setting as well, which suggests that the findings of Study 1 and 2 are ecologically valid.

### Relationship Between Implicit Theories and Learning Environments

In summary, the results support our hypotheses about the different effort allocation strategies between incremental and entity theorists. In a learning environment where they are allowed to choose a task out of many options, entity theorists tend to perform at least as well as incremental theorists. When there are multiple-task options, their aptitude exploration strategy may be an effective way to achieve a high outcome. This is a positive aspect of entity theorists, which has not been focused on in previous studies on a single-task situation. However, the present findings do not contradict previous studies nor imply that having an entity theory is desirable. Rather, the adaptive implicit theory may depend on whether they can choose a task from a wider range of tasks. In a situation where an individual aims to master one specific task, holding incremental theory might be effective because the available strategy is restricted to the task mastery strategy. Conversely, in a situation where there are sufficient task options and opportunities to switch, entity theory might be as effective as incremental theory, because it is more likely to find the task you can perform well. The significance of the present research is that by comparing the way tasks are provided in educational situations, it is possible to treat the environmental factor as a determinant of individuals’ beliefs about abilities.

### Implication for Cultural Difference on Implicit Theories

The present study had several limitations. First, although participants in Studies 1 and 2 were encouraged to perform well, they were not given incentives. Therefore, it is difficult to interpret their intention with a particular strategy. A follow-up experiment should be conducted with incentives for high performance. Second, in both experiments, participants had only one chance to switch their tasks. Some might have felt it was risky to engage in a new task with no information. In future research, we should consider controlling for individual differences to avoid uncertainty. Third, in Study 3, we only asked respondents to recall their past school experiences. Longitudinal surveys should be conducted to further investigate the effects of learning environments and implicit theories on performance.

Despite these limitations, the present research suggests a possible explanation for cultural differences in prevailing implicit theories. It is known that Japanese people are more likely to hold an incremental theory than American people (Heine et al., 2001; Church et al., 2012). This could be due to differences in the task structures of the two countries. Life in Japan features many situations in which people do not have a wide range of alternatives; for instance, the public school system in Japan is highly standardized, in which all students learn the same subjects and are evaluated using the same criteria. Similarly, regarding university admissions, they seldom consider an applicant’s abilities in areas beyond their entrance exam score. In other words, in Japan, the scholastic ability is assessed in a more one-dimensional manner compared to the United States (US; Tsukada, 1993; Arai, 2003). Under this system of educational evaluation, those who are mastery-oriented and direct their efforts toward a given task will more likely succeed. In contrast, many schools in the United States adopt the premise that ability varies and provide multiple curricula in which each student might excel (Tsuneyoshi, 1992, 2008). Under the United States system, exploring one’s aptitude in various spheres is a more reasonable attitude. In fact, it has been found that the correlation between implicit theories and academic performance differs among several countries (Costa and Faria, 2018; Jia et al., 2021). A different social system could make different implicit theories more adaptive in the culture. The relation between the ways in which tasks are provided in education and implicit theories might be deep. Therefore, it is necessary to investigate the relationship between educational environments and individual beliefs from a socio-ecological perspective.

## Data Availability Statement

The raw data supporting the conclusions of this article will be made available by the authors, without undue reservation.

## Ethics Statement

The studies involving human participants were reviewed and approved by The Research Ethics Committee of the Department of Social Psychology, The University of Tokyo. The patients/participants provided their written informed consent to participate in this study.

## Author Contributions

This work was conceived and designed by KS (Study 2 and 3) and NA (Study 1), under the supervision of YM. KS analyzed all data. KS and YM interpreted the results and wrote the manuscript. All authors contributed to the article and approved the submitted version.

## Funding

This work was supported by JSPS Grant-in-Aid for Scientific Research (C), Nos. 15K04024 and 19K03189 for YM.

## Conflict of Interest

The authors declare that the research was conducted in the absence of any commercial or financial relationships that could be construed as a potential conflict of interest.

## Publisher’s Note

All claims expressed in this article are solely those of the authors and do not necessarily represent those of their affiliated organizations, or those of the publisher, the editors and the reviewers. Any product that may be evaluated in this article, or claim that may be made by its manufacturer, is not guaranteed or endorsed by the publisher.

## Footnotes

^1^The post-task questionnaire contained items on the participants’ intentions, feelings, and reactions during and after the practice trials. The items measured the participants’ kanji knowledge, vocabulary, and experiences of being evaluated by parents and others, and of extracurricular activities. Those details are beyond the objective of this article, as these items were not used in the analyses.

^2^Throughout the studies, *b* represents the coefficients and Î²Î² represents the standardized coefficients. Standardized coefficients of the tobit regression analysis were calculated by HAD (Shimizu, 2016).

^3^The translation of the word “intelligence” was slightly different from the items used in Study 1.

^4^As in Study 1, we will provide the information of the other post-task questionnaire in the Supplementary Material.

^5^In Study 2, we mean-centered the variables to avoid the problem of multicollinearity, which we should consider while introducing interaction as an independent variable (Aiken and West, 1991).

^6^Simple slope analysis was conducted through the website of Preacher et al. (2006; quantpsy.org).

^7^In Japan, it is common that students enter high school through entrance exams or interviews unless they are in combined junior high and high school. The relative ranking of high schools is made public by indicating the likelihood of success as a deviation value.
