# Supplementary material for: Effect of Implicit Theory on Effort Allocation Strategies in Multiple Task-Choice Situations: An Investigation From a Socio-Ecological Perspective
Source: Front Psychol. 2021 Dec 3;12:767101. doi: 10.3389/fpsyg.2021.767101 (PMC8678568; doi:10.3389/fpsyg.2021.767101)
Supplement: Supplementary file 1 [file Data_Sheet_1.pdf]

Supplementary Material

Effect of Implicit Theory on Effort Allocation Strategies in Multiple Task-Choice Situations: An Investigation From a Socio-Ecological Perspective

Keita Suzuki, Naoki Aida, Yukiko Muramoto

1 Study 1

1.1 Post task questionnaire

- How did you feel when you were doing the social sensitivity (or metaphysical reasoning) task? For each of the following statements, please circle the number of the most appropriate option (1. Strongly Disagree ~ 6. Strongly Agree).

1 I focused on getting more money.
2 I focused on getting good grades.
3 I focused on improving my ability.
4 I focused on mastering new ability.
5 I tried to judge which task is right for me.
6 I tried to concentrate on the practice.
7 I concerned about what the other task would be like.
8 I tried to practice as long as possible.
9 I had fun.
10 I was frustrated.
11 The task was interesting.
12 The task was hard to bear.
13 I got tired of the task.
14 I think I got a good grade.
15 The task was difficult.
16 The task was easy.
17 I think I will be able to do the task to some extent with practice.
18 The task is not something that can be improved with practice.
19 My performance in this task depends on my knowledge of kanji (Chinese characters).

20 I think my knowledge of kanji is high.
21 I am confident in my vocabulary.
22 I think this task is measuring my social sensitivity.
23 I think this task is measuring my metaphysical reasoning ability.
24 I am confident in my social sensitivity.
25 I am confident in my metaphysical reasoning ability.

■ For each of the following questions about yourself, please circle the number of the most appropriate option (1. Definitely Effort ～ 6. Definitely Ability).

1 When you succeed in something, which do you want to be recognized for, your effort or your ability?
2 When you succeed in something, which do you want to show off, your effort or your ability?
3 When someone succeeds in something, which do you want to give credit for, his/her effort or ability?
4 When you succeeded in something in your childhood, which did your parents give credit for, your effort or your ability?
5 When you failed in something in your childhood, which did your parents point to, your lack of effort or your lack of ability?

■ What kind of lessons have you taken before? Please indicate the content of your lessons and the approximate length of time you have you been taking them.

■ What are your reasons for working hard in academic situations? For each of the following statements, please circle the number of the most appropriate option (1. Strongly Disagree ~ 6. Strongly Agree).

1 I don't want my friends to make fun of me.
2 I can learn new things.
3 I want to be noticed by my friends.
4 It is fun to master something.
5 I don’t want to be scolded by my parents and teacher.
6 I think what I am learning now is useful for my next learning.
7 I can show off to others when I get good grades.

8 I can build up my abilities by making effort.
9 I feel good when I beat my rivals.
10 I can improve my abilities.

2 Study 2

2.1 Post task questionnaire

■ How did you feel when you were doing the task? For each of the following statements, please circle the number of the most appropriate option (1. Strongly Disagree ~ 6. Strongly Agree).

1 I focused on getting good grades.
2 I focused on fulfilling myself by completing a difficult task and so on.
3 I intended to get good scores by engaging in the same task for a long time.
4 I intended to get good scores by choosing a task that I was suited for.
5 I was too late to switch tasks because I was stuck with the first one.
6 I decided to switch tasks because I got bored with the first task.
7 I felt joy in answering the task correctly.
8 I felt joy in working hard on the task itself.
9 I think the more I do task, the more I will be able to do it.
10 I had fun when I was solving the task.
11 I was frustrated when I was solving the task.
12 The task was interesting.
13 The task was hard to bear.
14 I got tired of the task.
15 I felt helpless while solving the task.

3 Study 3

3.1 The details of the questionnaire

3.1.1 Questions measuring the respondents’ educational experiences

Here, we describe the items used to measure the respondents’ experiences in elementary school. The same items were repeatedly used to measure the participants’ experience of elementary and high school as well (The words written in italic were replaced with “junior high school” or “high school”).

- Please tell us about classes at your elementary school.
  Please indicate how true you feel each statement to be by choosing the appropriate option. (1. Strongly Disagree ~ 6. Strongly Agree)
  1. At my *elementary school*, all students were expected to learn at the same pace.
  2. At my *elementary school*, learning was tailored to the individual through ability-based learning groups (such as grade-based class allocation).
  3. At my *elementary school*, delayed learning made school life uncomfortable.
  4. At my *elementary school*, many classes involved memorizing textbook content.
  5. At my *elementary school*, there were many classes which involved considering and researching topics that had been decided individually or in groups.
  6. At my *elementary school*, improvement in subjects with poor achievement was expected.
  7. At my *elementary school*, improvement in at least one subject with good achievement was expected.

- Please tell us about your study and school experience.
  Please indicate how true you feel each statement to be by choosing the appropriate option. (1. Strongly Disagree ~ 6. Strongly Agree)
  1. In *elementary school*, I studied focusing on subjects featured in future entrance exams.
  2. In *elementary school*, I tried to find and develop my talents, not just in my studies.
  3. In *elementary school*, I tried without giving up when facing difficulty.
  4. In *elementary school*, I believed that ability is something about you that you can't

change very much.

- Please tell us about your attitude to *elementary* school.
  Please indicate how true you feel each statement to be by choosing the appropriate option. (1. Strongly Disagree ~ 6. Strongly Agree)
  1. I was almost never late at school.
  2. I often fell asleep in class.
  3. I was satisfied with my grades.
  4. I was satisfied with my friendships.
  5. Overall, I was satisfied with my *elementary school* experience.

- When you were in your final grade of the *elementary school*, how was the relative ranking of your academic record? Please indicate the appropriate option.
  1. Among the highest
  2. Relatively high
  3. Around the middle range
  4. Relatively low
  5. Among the lowest
  6. None of those are appropriate

3.1.2 Additional questionnaires used to measure the information of respondents' high school

- Did you go through an entrance exam or an interview before entering the high school?
  1. Yes
  2. No

- Were you in a combined junior high and high school?
  1. Yes
  2. No

■ How was the relative ranking of your high school among the high schools in Japan? Please indicate the appropriate option.

1. Among the highest
2. Relatively high
3. Around the middle range
4. Relatively low
5. Among the lowest
6. None of those are appropriate

3.2 Analysis with the variables of elementary and high school

We conducted a parallel analysis with Model 1-1, 1-2, in which the implicit theory, uniformity of education and the academic record of junior high school was replaced with those in elementary school and high school (Table 1).

When the variables of elementary school were used, the interaction of implicit theories and uniformity of the education was significant (Model 3-1:$\beta = -.094, p = .008$, Model 3-2: $\beta = -.078, p = .035$). We conducted a simple slope analysis on Model 3-2 (Figure 1-(1)). Among the participants whose uniformity of the education was high (+1SD), the main effect of implicit theories was significant ($\beta = -.243, p < .001$), suggesting that the more participants endorse incremental theory, the higher the relative rank of the academic record will be. Among the participants whose uniformity of the class was low (-1SD), the main effect of implicit theories was not significant ($\beta = -.088, p = .194$).

When the variables of high school were used, the interaction of implicit theories and uniformity of the education was not significant (Model 4-1:$\beta = .018, p = .605$, Model 4-2: $\beta = -.013, p = .730$) (Figure 1-(2)).

The result of the analysis of using the variables of elementary school was consistent with Model 1-2 and 2-2. On the other hand, when the variables of high school were used, the moderative effect of uniformity of education was not found.

3.3 Figure 1

(1) Elementary school

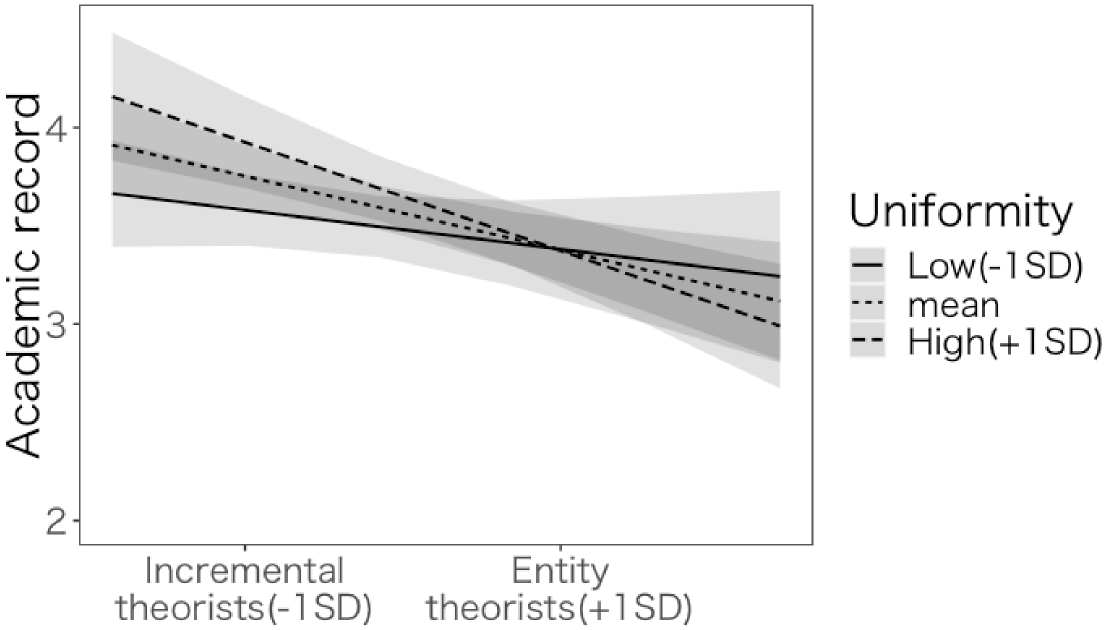

(2) High school

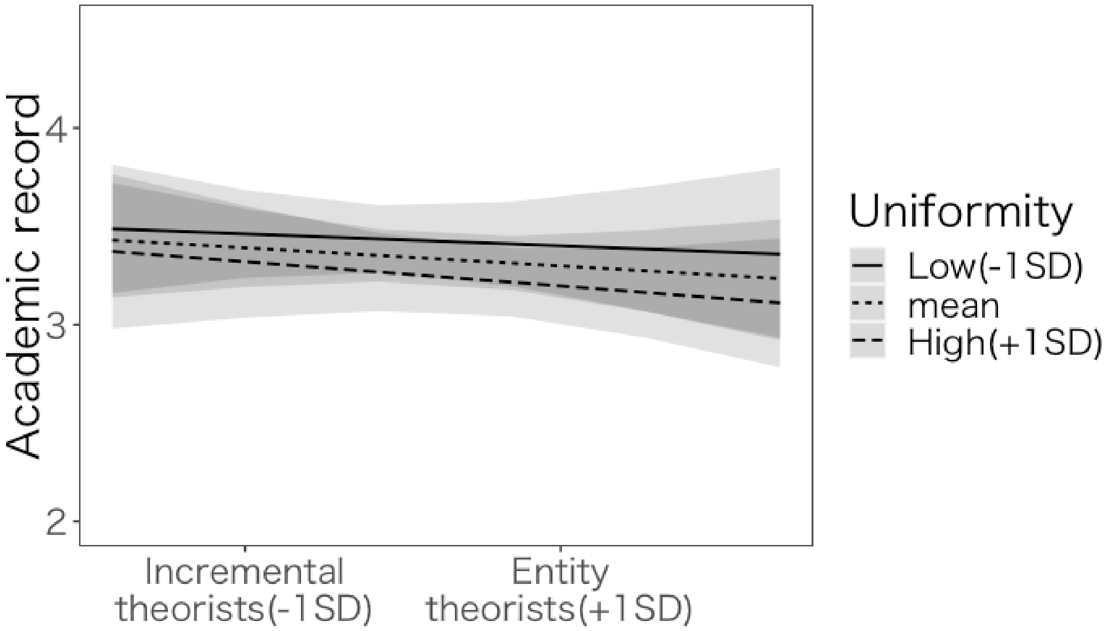

Figure 1. The effect of implicit theories and Uniformity of the education on academic record in (1) elementary school and (2) high school.

3.4 Table 1. Results from regression models on the academic record

Independent Variables	Model 3-1 Academic record in elementary school (N = 452)		Model 3-2 Academic record in elementary school (N = 374)		Model 4-1 Academic record in high school (N = 458)		Model 4-2 Academic record in high school (N = 382)	
	β	t	β	t	β	t	β	t
Implicit theories	-.146	-2.95**	-.164	-3.15**	-.041	-0.84	-.038	-0.70
Uniformity of education	.069	1.39	.074	1.41	-.008	-0.16	-.077	-1.37
Implicit theories × Uniformity of education	-.094	-2.66**	-.078	-2.11*	.018	-0.52	-.013	-0.35
**Covariates**								
Educational attainment dummy (Father; 1 = Graduated university)			.223	4.21**			.073	1.32
Educational attainment dummy (Mother; 1 = Graduated university)			-.025	-0.49			-.083	-1.54
Economic status			.053	1.07			.153	2.95**
Age			-.019	-0.38			.064	1.24
Gender			-.010	-0.21			-.021	-0.41

**$p < .01$, * $p < .05$, †$p < .10$
